# Indents

**Published:** 1909-05-15

**Authors:** 


### INDENTS.

☞ **Brief Articles of Technical or Scientific Value will receive notice and comment. Contributions invited.**

*To Stop Hiccough.* Have the patient take a piece of sugar dipped in vinegar and ask him to chew it rapidly and swallow, which will immediately stop the hiccough. This simple method never fails even in case of hysterical hiccough, which yields to no other treatment.—*Annales Dentaires.*

*Pressure Anesthesia.* In applying pressure anesthesia through the opening made or otherwise, and without any special instrument or syringe, follow these instructions: Bore a hole with a small bur through the enamel at some convenient point, preferably at the neck of the tooth. With a bit of cotton fill the hole loosely, and then saturate the cotton with the cocain solution and keep it protected from saliva. Then stick a small ball of gutta-percha on the broad flat face of an amalgam plugger and, warming just a trifle, push it down over the hole as hard as you can. After a moment the hole may be drilled deeper, and if sensitivity is reached, the operation must be repeated as often as necessary.

On reaching the pulp an exploration will quickly determine whether it is numb all through or not; if not the same *modus operandi* may be repeated, carrying the anesthesia as much further as required.—R. B. Tuller, *Dental Digest.*

*Neuralgia.* The important thing is to find the organ or disease causing the mischief, and, where possible, remove the cause. A careful study of the area of the pain and tenderness will help to elucidate the cause. Still, there will be cases where no definite cause can be found.

The next in importance will be to treat the general condition of the patient by placing him under the most suitable hygienic conditions and giving such treatment as the general condition of the patient may indicate.

The antipyrin group, in particular phenacetin, have a very beneficial palliative effect in all cases of referred visceral pain. Gelsemium and butyl chloral are very useful in all cases of trigeminal neuralgia.

*Local Treatment.*—Hot application, sometimes cold, will generally be comforting and will serve to protect the parts from draughts, etc.

*Sedative Application.*—Menthol dissolved in chloroform, with aconite; or, aconite and belladonna is very useful. Lead and opium may be tried.—Wm. Mattews in *American Journal.*

*Danger in Handling Hydrofluoric Acid.*

Your warning in a recent editorial on the dangers of handling hydrofluoric acid leads me to request publication of my experience with this drug. In pouring out the acid from the wax bottle into a small platinum dish wherein I was accustomed to place gold inlays recently cast, to clean them, I accidentally got a little of the acid under a fingernail. The pain was almost unbearable, and continued to become worse, although medical aid was sought and a number of different remedies tried. In looking over my medicines for a remedy I finally sighted a can of antiphlogistine, and as it promised at least a cooling effect for a time, the finger was thrust into this. The pain ceased almost instantly and did not recur. The finger was kept in this for about ten minutes. Subsequently a darkened spot showed under the nail, indicating where the acid had taken effect, but no further trouble has occurred. I now keep the acid in a wide-mouthed hard rubber jar with a screw cap—a traveler's inkstand I believe it is—and drop my inlays into that until the desired effect has been obtained.—E. S. Fuller, in *Cosmos.*

*Another Chloroform Fatality.* The *Frederick News* of Frederick, Md., for February 24th, 1909, publishes an account of the death of a patient, a woman thirty-three years of age, who expired suddenly in a dentist's chair while undergoing an operation for the extraction of teeth under chloroform narcosis.

We had supposed that the danger of using chloroform as a general anesthetic for the operation of tooth extraction was sufficiently well known, and that the record of chloroform fatalities in the dental operating chair was sufficiently large, to have prevented a further repetition of the foolhardiness of using chloroform under these circumstances.

When the bad record of chloroform anesthesia for dental operations is considered on the one hand, and when the practical safety of nitrous oxide is considered on the other hand, the use of chloroform anesthesia in dentistry seems inexcusable. The fact that the anesthetic in the Frederick case was administered by a physician simply shifts the responsibility, but does not in the slightest degree mitigate it. —*Cosmos.*

*Have a Program.* Every dentist should make a mental program of his day's work for the first thing in the morning and then make a business of accomplishing this work. Such a plan helps to keep the work moving and each day sees definite progress made. A dentist should not permit his work to accumulate, even though he be compelled to work late in the evening. "Do it now," is an excellent motto to stimulate one when he begins to lose interest in his work. When you begin a piece of work complete it. Don't let it drag along or permit patients to delay work. Finish it as soon as possible, and the collections are more easily made.

See to it every morning that you are in perfect tune with yourself and the world before you ever leave your home for the office. Mental discord is fatal to quality in work. The man who goes to work feeling out of sorts with everybody is in no condition to do the taxing work required of a dentist. Did you ever notice how, when you are in harmony, you feel like a giant; then it is that you are ready to meet the duties of the day? —Dr. O.U. King in *Dentist's Record.*

*Is Saliva an Antiseptic?* This question has been brought into prominence in discussing the why and wherefore of some mouths being immune to dental caries. No evidence has been found to support the theory that this is due to any antiseptic qualities in the saliva. The more recent research intimates that immunity is due to the saliva being deficient in food constituents upon which the microbe of dental caries thrives. This condition cannot be brought about by local prophylaxis, or by drugs alone. It is a normal condition, and its non-existence is evidence that the nutritive functions are not normal, although not necessarily pathological. The remedy is to be found by a careful investigation of the food intake and the waste output, and a change brought about by a carefully directed diet, aided in the onset by temporary drug treatment. The normal condition can be maintained only by right living and right feeding; what constitutes this must be determined in each individual case. This form of malnutrition is quite compatible with excellent general health, and does not of itself shorten life, as is evidenced by its many victims reaching advanced age in excellent physical and mental condition after being many years toothless.—Trueman in *Brief.*

*Salicious Cement.* There is one unfortunate quality about this material—it is not very sticky. It is not very friendly to tooth-substance, so that we have to prepare cavities as for gold fillings, with undercuts, etc. In view of the fact, I have recently experimented a little in the use of the oxyphosphate in combination with this new material, taking advantage of the sticky property of the oxyphosphate, in order to retain the fillings without having to make quite such deep undercuts. I mix the cement at the same time, as nearly as possible, and put the oxyphosphate in the bottom of the cavity, and then quickly knead the new filling into that, working it in and hoping thereby to get a hold upon the surface of the cavity that I cannot get with the new cement alone. In finishing large cavities on approximal surfaces that came well into the grinding surface, I have in a number of instances filled the cavity with silicate and then cut out a portion and covered with amalgam, with a view of securing a better protection, and as far as I have observed they look very well. Amalgam protects by covering the surface exposed to stress. It does not leave little edges exposed, the edges along the buccal and lingual sides remaining very smooth. This is an advantage over an entire amalgam filling.—Dr. Perry in *Cosmos*.

*Non-Corossive Instruments.* Last year Dr. Ames suggested the use of some kind of varnish on steel instruments to be used in manipulating the silicate cements. He suggested a liquid court plaster known as New Skin. I have had for a good many years in my medicine closet one or two bottles of varnish that I obtained from a jewelry supply house years ago when experimenting to find a transparent inlay setting material. It is sold as liquid amber. The manufacturer, Bruce Murphy, of Orillia, Can., wrote me at that time that it really is a solution of amber in ether. It has a strong odor of ether and appears like amber. He claims to have some secret process by means of which he dissolves the amber in ether.

I presume many of you have found that the instruments supplied for manipulating these cements are so thick and clumsy that they are unsatisfactory for reaching the places when we want to use them.

Following Dr. Ames' suggestion, I began to use varnished steel spatulas for this work. After each use, my secretary cleans them and dips them into the bottle and lays them aside for two or three hours in order to let them dry, when I

find them coated with a very thin and very adhesive coating of this varnish. These spatulas work well in manipulating the cement and forcing it into places of difficult access.

*Suggestions for Mucin Tests.* Try the following simple and suggestive experiment:

When the opportunity next presents, collect four or five C. C. of thick, viscid, ''ropy'' saliva and divide equally between four or five test tubes. In so doing, note how glutinous and tenacious it is. You may need shears to cut it .

Set the tubes aside while you proceed to scrub the teeth with orangewood stick and pumice. Note the frictionless, slimy coating and the amount of scouring required before the pumice laden stick penetrates to the enamel and produces that characteristic clean tooth feeling.

Now return to the tubes. To the first add eight or ten drops of acetic acid and one C. C. of water. Agitate and note the large amount of mucin present. Each of the other tubes contains a like amount. To the second tube add one C. C. of glyco-thymoline, borol, or any other preparation containing coloring matter, and one C. C. of alcohol. Note the three distinct layers. The glyco-thymoline below and the alcohol above would mix instantly if they could get together, but note how effectually they are separated by the saliva. Note also the gradual abstraction, by the alcohol, of the water from the saliva, and the effect on the mucin. Note the time required before the three liquids will unite readily.

To the remaining tubes add to one an equal quantity of water; to another a saturated solution of bi-carbonate of soda, to another a straw colored solution of tincture of iodine, or any other preparation you may be using. Note the changed relative positoins of the saliva and the water when the water is free and when it contains some agent in solution and note also the degrees of readiness or unreadiness with which the saliva and the different solutions mix.

Having noted these things and observed that such saliva is not readily miscible, recall that beneath the slime, upon the surface of the teeth, innumerable colonies of acid producing bacteria are comfortably housed, raising families and increasing their output. Then reflect upon the probability of the so-called antiseptic mouth washes, as ordinarily employed, penetrating that slime and evicting them or even neutralizing to any practical extent, the end products of those industrious but undesirable citizens.—Dr. Bowles in *Odontoblast.*

*Capping Exposed Pulps.* In the writer's practice, all of the oft-tried materials placed in contact with the exposure have been discarded in favor of the "Iodoformagen Cement" which has given some phenomenal results in many "forlorn hope" cases where the condition of the patient compelled the operator to make an attempt on the one frail chance of success with the knowledge that if the pulp would not live it would "pass away quietly and peacefully."

To insure success certain rules of procedure must be observed.

No drug of any kind other than the mild alkaline solution should be employed, not even an antiseptic. The Iodoformagen will take care of that.

The mixture should be of such consistence that it will flow. This mixture should be placed on a disk of asbestos felt large enough to well cover the exposure. This disk should be previously saturated with the Iodoformagen liquid. The disk should then be carefully slid into place over the exposure without pressure.

The paste may be mixed ready for use before applying the rubber dam as it will remain plastic on the slab for some time but it hardens almost immediately upon introduction into the cavity, turning green, thus indicating some chemical reaction.

If the exposure be in the line of pressure in the introduction of the filling, a thin platinum or gold disk should be placed over the asbestos, coating the cavity side of the metal disk with the paste.

In deep cavities the capping should be covered with Original Oxyphosphate of Copper. Indeed this material may often replace the metal disk. Where its color is not objectionable the whole cavity may be filled with it, allowing it to remain as long as it maintains its bulk. If a metal filling is still contra-indicated the surface of the filling may be replaced with the more enduring "New Process." Under no condition should a metal filling of any kind be placed at the same series of sittings in which the capping is attempted.

The whole plea of this article is based upon a recognition of the fact that the cutting necessary for the placing of the so-called "permanent" fillings often disturbes processes of nature designed to combat disease or awakens activities which may result disastrously.

That time and rest are two of the most important factors in the cure of diseased conditions.

Let us study Nature's processes more carefully and in our therapeutic and technical procedures endeavor to act as her able ally instead of defeating her by well intentioned but illadvised interference.—Dr. T. E. Weeks in *Garretsonian*.

*Dental Research.* There is in New York City, at Sixty-sixth street and Avenue A, an institution established by a fund supplied by Mr. John D. Rockefeller, called the Rockefeller Institution of Medical Research. In a communication from Dr. Simon Flexner in charge, it is stated that there is at present no one on the staff interested in the dental field; but that such a line of work is by no means excluded. The staff is now entirely occupied with other matters; but the reason that dental problems are not taken up is not because they do not realize the importance of this particular work.

Most of the work done at this institution is published in the Journal of Experimental Medicine, which is issued bimonthly at a subscription price of $5.00 per year.

Now, since dental problems, concerning the medical profession at large as a branch of medicine, and incidentally and yet directly concerning humanity at large, are not excluded from the work of this already established institution, it might be inferred that if some concerted and interested action was taken by the dental profession through their societies, there might be brought about the ways and means of establishing a chair in this institution, with ample facilities (in conjunction with what is already being done) for the investigation and research so much needed in the dental branch of medicine; carrying with it as it would, important benefits to the entire field of medicine as well as to the dental specialist and to the world.

Considering the fact that the oral cavity as the vestibule of the alimentary tract, through which normal nourishment, and largely also the breath of life enters, and that the teeth and fluids of the mouth are prime factors in the preparation of the food passing into the system, and have an important influence on digestion and metabolism, it would seem that this part of our organism, the alpha of human physiological phenomena, so to speak, should have first place in these matters of medical research, and especially deranged action at that point, that may and do lead to other and often serious derangements of the whole human system.

This is a matter that should interest us all, and with the largest local dental society in the world in Chicago (over 1,000 active members), a component of the state society, whose endorsement could be counted upon (as well as the concurrence of the entire profession), this would seem to be a good place to agitate the matter, and set on foot a movement that should bear good fruit. The establishment of a dental chair in an institution already organized and operating in so-called research, and its maintainance assured by ample endowment, is a matter that should not be allowed to go by default, if the right active interest has the least show of bringing about the desired end.—R. B. Tuller in *American Journal.*

*Proprietary Preparations.* That the habit of recommending and prescribing proprietary nostrums is one that has invaded, not only the medical but the dental profession, is a potent fact. Particularly is this true in regard to mouth washes, and many and varied indeed are the schemes evolved to provide an alluring bait for the unsuspecting professional man. Who is there among us that has not been profferred the opportunity to get "in on the ground floor" by purchasing dividend paying stock, the small financial outlay to secure an annual pittance during old age? The only and essential requirement of the dentist in such cases being that he fill in parrot like, the name of the patient on neatly printed prescription pads furnished by the manufacturers of the much vaunted "antiseptic wash."

A new Detroit company, incorporated under Arizona laws, and capitalized at five million dollars, is the latest to introduce a liquid antiseptic to the public through the dental profession. But its field of usefulness is not confined to the mouth alone. According to the handsomely embellished label, it not only prevents pyrrhœa but removes tartar deposits, tan, sunburn and moth patches, thus enabling the dentist to become a beauty specialist as well. Or, should he betimes be called upon to embark in the tonsorial art, he will again—according to the label—find it a luxury after shaving. The label still further informs us, however, that they are but the distributors—mark you DISTRIBUTORS—of the liquid.

But it is not of the merits of the preparation we wish to make note, rather the method of introduction that the company has assumed. It surpasses former promoters inasmuch as it gives absolutely free of charge, one thousand shares of stock—par value, one thousand dollars—to any dentist prescribing twelve bottles per month for sixteen months, and in addition thereto agrees to pay the dentist at the expiration of that time twelve percent. on all goods prescribed.

Leaving out of our discussion the ethical side of the question, the proposition upon first appearance is naturally an attractive one from a financial viewpoint, but does not bear closer inspection. If sufficient dentists can be secured to prescribe and recommend their goods during sixteen months the company would be in a flourishing condition at the end of that time, but what assurance have the stockholders that the manufacturers will continue to permit them to act as ''distributors'' after the product is fairly introduced? It is not only reasonable to suppose that they will act as their own distributors, and if so of what value is the stock?

Again we are not advised as to who are the promoters or what over worked genius after years of experiments discovered this wonderful formula, but if the word of their representative is to be taken it bears a striking resemblance, both in ingredients, color and flavoring to the Liquid Antiseptic as published in the National Formulary, a preparation that is universally prescribed by physicians and dentists.—*Odontoblast.*

*Curing Pyorrhea.* The question as to whether pyorrhea alveolaris is a local or a systemic disease is one which has been discussed by the profession a great deal. I have the feeling that there are some cases which are due simply to local irritation because we know that in many instances the thorough removal of the deposits and proper treatment locally will result in a cure of the disease. That has been demonstrated many times. There are other cases, however, in which I believe I am not rash when I make the statement, that local treatment, no matter how skillfully performed, or conscientiously carried out, will not result in the cure of the disease, and I

cannot be convinced that constitutional effects are not a great factor in many cases of this disease. I had one instance I should like to recite which goes to prove the fact that systemic tendencies are sometimes a very important factor. I had a lady patient whose teeth were becoming loose; in fact, two of the upper molars were lost from this disease. The lower molars were evidently going the same way. I gave them the ordinary treatment as skillfuly as I could, but I could not be as thorough in the instrumentation as I should have liked because of the sensitiveness of the tissues and the teeth themselves, so I considered the work I did was not thorough.

She went away on a vacation; the lower molars were quite loose when she went away, and I felt I could not accomplish very much for her. She came back in the fall, not having seen a dentist in the meantime. She looked better physically, and when I examined the teeth I found them comparatively firm and the gums around them in good condition, so much so that I asked her if she had had any treatment, and she said she had not. I think undergoing systemic treatment in the way of regulation of the diet, etc., had something to do with the improvement. I saw her two weeks ago and those teeth are absolutely firm today and the gums normal, and the pockets are closed where formerly I could probe at least half way down the roots of the teeth. It was not my treatment that did it, because I have treated other cases more skillfully, more carefully, and with better instrumentation than I was able to give in that case, and I have failed utterly to accomplish any result such as that; and so while local treatment is exceedingly important, and while I do not believe systemic treatment will cure these cases when there are extensive and deep deposits, at the same time I cannot be convinced that constitutional effects do not have a very great influence upon this disease in many cases of its features.

As to the local treatment, most of us are not thorough enough when we attempt to treat a case of this kind. It is a long tedious operation to go through a mouth in which there are many pyorrhea pockets, and properly operate upon them. It is not a simple or easy matter to remove the deposits. But that is not all. It is going beyond that and smoothing down the surface of the root that is hard and flint like until it is not only smooth, but until the apparently hard incrustation is removed and we get down to good tissue into which the scaler will bite easily. We do not ordinarily follow down to the bottom of these pockets and give the instrumentation which will result in an absolute obliteration of all of that necrotic tissue and the stimulation of new granulations to fill the pockets, and unless we do that we are failing in our local treatment. I believe with the essayist that local medication of these cases is not as important a factor in the treatment of this disease as a great many believe. I believe in the thorough removal of the deposits, if this can be done, but we cannot always do it.

The treatment of this disease has been in my hands one of the most difficult and most unsatisfactory factors of my practice, although I have had some very encouraging results in treating pyorrhea. However, I cannot get results in this disease that I feel I can in other diseases. I can take a chronic alveolar abscess, I can take a badly broken-down tooth, and approach that kind of operation with a great deal more confidence and more satisfaction than I can a case of pyorrhea alveolaris. And still there is this feature of it I want to impress upon the profession generally, and that is, a large number of dentists are failing in their duty to patients when they consult them for relief of this trouble. Many of them, if they see pus coming from a pocket, will say it is pyorrhea alveolaris, and that they cannot do anything for those cases. That is a cowardly position for any dentist to take. He can stop that pus, and he ows it to his patients to do so, and while I cannot promise to cure cases of pyorrhea alveolaris in all instances, I can promise patients to make their mouths healthier than when they came to me. I do not think any man ought to promise to cure every case of pyorrhea alveolaris that comes to him,because I do not believe he can do it.—Dr. C. N. Johnson in *Review*.

